# Supplementary material for: Can We Safely Obtain Formal Oxidation States from Centroids of Localized Orbitals?
Source: Molecules. 2020 Jan 6;25(1):234. doi: 10.3390/molecules25010234 (PMC6983110; doi:10.3390/molecules25010234)
Supplement: Supplementary file 1 [file molecules-25-00234-s001.pdf]

molecules

MDPI

Supporting Information

Can we obtain formal oxidation states from centroids of localized orbitals?

Martí Gimferrer, Gerard Comas-Vilà and Pedro Salvador*

Institut de Química Computacional i Catàlisi i Departament de Química, University of Girona, Maria Aurèlia Capmany 69, 17003 Girona, Spain

* Correspondence: pedro.salvador@udg.edu; Tel.: +34-972418358

Table S1. Hydrides oxidation states (OS) from the EOS analysis, together with its reliability index R (%), and from the localized orbitals (PM and NLMOs) centroid. Electrons from the centroid assigned according to a) distance (closest atom (CA)) and b) atomic basin (BA).

Mol.	H (EOS)	R (%)	H (PM-CA)	H (PM-BA)[b]	H (NLMO-CA)	H (NLMO-BA)
LiH	1	100.0	-1	-1	-1	-1
BeH_2	2	100.0	-1	-1	-1	-1
BH_3	3	100.0	-1	-1	-1	-1
CH_4	4	46.2	-1	-1	-1	-1
NH_3	-3	81.0	-1	-1	+1	-1
H_2O	-2	100.0	-1	-1	+1	-1
HF	-1	100.0	+1	+1	+1	+1
NaH	1	100.0	-1	-1	-1	-1
MgH_2	2	100.0	-1	-1	-1	-1
AlH_3	3	99.8	-1	-1	-1	-1
SiH_4	4	97.9	-1	-1	-1	-1
PH_3	3	97.1	-1	-1	-1	-1
H_2S	-2	48.9	-1	-1	+1	+1
HCl	-1	100.0	-1	+1	-1	+1

Table S2. Structural and electronic parameters of the hydrides studied, including Allen's electronegativity (EN), bond distance, EFO occupancy, distance to the centroid (computed using the PM and NLMO localized orbitals) and distance to the bond critical point (*bcp*).

Atom	Mol.	EN	Bond Dist.	EFO Occ.	Cent-At. (PM)	Cent-At. (NLMOs)	*bcp*-H
Li	LiH	0.912	1.607	0.112	1.403	1.401	0.886
H	LiH	2.300		0.824	0.205	0.206	
Be	BeH_2	1.576	1.344	0.178	1.074	1.072	0.766
H	BeH_2	2.300		0.788	0.270	0.271	
B	BH_3	2.051	1.200	0.195	0.888	0.887	0.669
H	BH_3	2.300		0.71	0.312	0.312	
C	CH_4	2.544	1.097	0.391	0.727	0.727	0.395
H	CH_4	2.300		0.429	0.370	0.371	
N	NH_3	3.066	1.024	0.588	0.609	0.615	0.278
H	NH_3	2.300		0.278	0.416	0.408	
O	H_2O	3.610	0.973	0.729	0.511	0.530	0.200
H	H_2O	2.300		0.179	0.462	0.442	
F	HF	4.193	0.933	0.836	0.435	0.455	0.159
H	HF	2.300		0.122	0.498	0.478	
Na	NaH	0.869	1.898	0.17	1.585	1.582	0.892
H	NaH	2.300		0.76	0.313	0.316	
Mg	MgH_2	1.293	1.714	0.233	1.427	1.397	0.833
H	MgH_2	2.300		0.76	0.287	0.318	
Al	AlH_3	1.613	1.593	0.249	1.284	1.281	0.792
H	AlH_3	2.300		0.746	0.309	0.312	
Si	SiH_4	1.916	1.492	0.234	1.139	1.136	0.758
H	SiH_4	2.300		0.713	0.353	0.356	
P	PH_3	2.253	1.431	0.183	0.977	0.998	0.720
H	PH_3	2.300		0.653	0.454	0.433	
S	H_2S	2.589	1.353	0.427	0.845	0.871	0.479
H	H_2S	2.300		0.439	0.508	0.482	
Cl	HCl	2.869	1.291	0.604	0.738	0.765	0.365
H	HCl	2.300		0.307	0.553	0.526	

Table S3. Allen's electronegativity ratio (χ_X/χ_H), relative EFO occupancy (relOcc), distance to the centroid ratio and relative to the total (computed using the PM (1) and NLMO (2) localized orbitals) and relative distance to the *bcp* (a).

Mol.	(χ_X/χ_H)	relOcc	rCent-At (1)	rCent-At (2)	relDist. (1)	relDist. (2)	relDist. (1)[a]	relDist. (2)[a]
LiH<br>LiH	0.397	-0.761	6.858	6.801	-0.745	-0.744	-0.681	-0.680
BeH_2<br>BeH_2	0.685	-0.631	3.980	3.951	-0.598	-0.596	-0.496	-0.494
BH_3<br>BH_3	0.892	-0.569	2.851	2.840	-0.481	-0.479	-0.358	-0.357
CH_4<br>CH_4	1.106	-0.046	1.968	1.961	-0.326	-0.325	-0.025	-0.024
NH_3<br>NH_3	1.333	0.358	1.465	1.510	-0.189	-0.203	0.138	0.130
H_2O<br>H_2O	1.570	0.606	1.105	1.199	-0.050	-0.090	0.262	0.242
HF<br>HF	1.823	0.745	0.874	0.951	0.067	0.025	0.339	0.319
NaH<br>NaH	0.378	-0.634	5.068	5.007	-0.670	-0.667	-0.579	-0.576
MgH_2<br>MgH_2	0.562	-0.531	4.968	4.398	-0.665	-0.630	-0.546	-0.515
AlH_3<br>AlH_3	0.701	-0.499	4.155	4.105	-0.612	-0.608	-0.483	-0.480
SiH_4<br>SiH_4	0.833	-0.506	3.226	3.193	-0.527	-0.523	-0.405	-0.402
PH_3<br>PH_3	0.980	-0.562	2.151	2.305	-0.365	-0.395	-0.266	-0.287
H_2S<br>H_2S	1.126	-0.014	1.662	1.810	-0.249	-0.288	0.029	0.002
HCl<br>HCl	1.247	0.326	1.335	1.454	-0.144	-0.185	0.187	0.161

Table S4. Carbenes oxidation states (OS) from the EOS analysis, together with its reliability index R (%), and from the localized orbitals (PM and NLMOs) centroid. Electrons from the centroid assigned according to a) distance (closest atom) and b) atomic basin.

	OS EOS			OS (PM)[a]		OS (PM)[b]		OS (NLMOs)[a]		OS (NLMOs)[b]	
**Mol.**	**M**	**L**	**R (%)**	**M**	**L**	**M**	**L**	**M**	**L**	**M**	**L**
1	0	0	67.8	0	0	0	0	0	0	0	0
2	0	0	61.2	0	0	0	0	0	0	0	0
3	0	0	59.2	0	0	0	0	0	0	0	0
4	2	-2	56.9	0	0	0	0	0	0	0	0
5	6	-2	72.0	6	-2	6	-2	6	-2	6	-2
6	6	-2	74.1	6	-2	6	-2	6	-2	6	-2
7	6	-2	64.1	6	-2	6	-2	6	-2	6	-2
8	6	-2	66.9	6	-2	6	-2	6	-2	4	0
9	6	-2	65.7	6	-2	6	-2	6	-2	6	-2
10	4	-2	51.5	4	-2	0	2	0	2	-2	2
11	2	0	50.4	2	0	2	0	0	2	0	2
12	2	0	55.5	2	0	0	2	0	2	0	2
13	2	0	58.1	2	0	0	2	0	2	0	2
14	2	0	55.9	2	0	0	2	0	2	0	2
15	2	0	62.4	2	0	2	0	0	2	0	2
16	2	0	63.3	2	0	2	0	0	2	0	2

Table S5. σ and π EFO occupation and relative EFO occupancy of the M and L of the Fischer-type carbenes studied. LO = Lowest occupied, FU = First unoccupied.

	**M**		**L**			
**Mol.**	**LO (π)**	**FU (σ)**	**LO (σ)**	**FU (π)**	**σ relOcc**	**π relOcc**
1	0.343	0.194	0.760	0.131	-0.593	0.447
2	0.325	0.198	0.744	0.214	-0.580	0.206
3	0.328	0.187	0.766	0.236	-0.608	0.163
11	0.464	0.388	0.529	0.461	-0.154	0.003
12	0.494	0.438	0.511	0.439	-0.077	0.059
13	0.508	0.427	0.510	0.428	-0.089	0.085
14	0.504	0.429	0.515	0.444	-0.091	0.063
15	0.540	0.411	0.535	0.363	-0.131	0.196
16	0.544	0.401	0.534	0.361	-0.142	0.202

Table S6. σ and π EFO occupation and relative EFO occupancy of the M and L of the Schrock-type carbenes studied. LO = Lowest occupied, FU = First unoccupied.

**Mol.**	**FU + 1 (σ)**	**FU (π)**	**LO (π)**	**LO - 1 (σ)**	**σ relOcc**	**π relOcc**
4	0.214	0.284	0.354	0.711	-0.538	-0.109
5	0.299	0.337	0.557	0.611	-0.342	-0.246
6	0.301	0.324	0.566	0.606	-0.336	-0.272
7	0.333	0.384	0.526	0.598	-0.284	-0.156
8	0.338	0.368	0.538	0.593	-0.273	-0.188
9	0.333	0.379	0.535	0.595	-0.283	-0.171
10	0.404	0.454	0.469	0.530	-0.134	-0.016

Table S7. Structural parameters of the Carbenes studied, including bond distance and, for the σ-like LO, distance to the centroid (computed using the PM and NLMO localized orbitals) and distance to the bond critical point (*bcp*).

Atom	Mol.	Bond Dist.	Cent-At. (PM)	Cent-At. (NLMOs)	*bcp*-C
W	1	2.224	1.583	1.507	1.096
C			0.646	0.725	
W	2	2.136	1.489	1.424	1.047
C			0.647	0.713	
W	3	2.098	1.520	1.408	1.026
C			0.577	0.689	
W	4	2.069	1.387	1.295	0.999
C			0.682	0.774	
W	5	1.917	1.134	1.091	0.880
C			0.795	0.835	
W	6	1.922	1.105	1.073	0.884
C			0.819	0.851	
W	7	1.900	1.164	1.065	0.863
C			0.753	0.845	
W	8	1.903	1.088	1.037	0.866
C			0.817	0.867	
W	9	1.916	1.119	1.063	0.874
C			0.806	0.861	
Os	10	1.837	0.962	0.916	0.814
C			0.878	0.924	
Os	11	1.834	0.954	0.920	0.812
C			0.881	0.916	
Ru	12	1.804	0.933	0.870	0.792
C			0.873	0.936	
Ru	13	1.801	0.923	0.887	0.791
C			0.881	0.916	
Ru	14	1.828	0.941	0.858	0.808
C			0.886	0.970	
Ru	15	1.851	0.989	0.935	0.831
C			0.864	0.918	
Ru	16	1.851	0.982	0.937	0.831
C			0.871	0.917	

Table S8. Structural parameters of the Carbenes studied, including bond distance and, for the π-like LO, distance to the centroid (computed using the PM and NLMO localized orbitals) and distance to the bond critical point (*bcp*).

Atom	Mol.	Bond Dist.	Cent-At. (PM)	Cent-At. (NLMOs)	*bcp*-C
W	2	2.136	0.239	0.234	1.047
C			1.899	1.903	
W	3	2.098	0.329	0.364	1.026
C			1.769	1.734	
W	4	2.069	0.565	0.595	0.999
C			1.504	1.473	
W	5	1.917	1.187	1.155	0.880
C			0.731	0.762	
W	6	1.922	1.206	1.194	0.884
C			0.716	0.728	
W	7	1.900	1.103	1.082	0.863
C			0.797	0.819	
W	8	1.903	1.107	1.097	0.866
C			0.796	0.807	
W	9	1.916	1.234	1.195	0.874
C			0.684	0.722	
Os	10	1.837	0.925	0.888	0.814
C			0.913	0.950	
Os	11	1.834	0.886	0.876	0.812
C			0.948	0.959	
Ru	12	1.804	0.845	0.809	0.792
C			0.959	0.995	
Ru	13	1.801	0.806	0.799	0.791
C			0.996	1.003	
Ru	14	1.828	0.836	0.825	0.808
C			0.991	1.003	
Ru	15	1.851	0.730	0.783	0.831
C			1.123	1.069	
Ru	16	1.851	0.735	0.734	0.831
C			1.120	1.119	

Table S9. Centroid distance ratio and relative to the total (computed using the PM (1) and NLMO (2) σ localized orbitals). Relative distance to the *bcp* (a).

At.	Mol.	rCent-At (1)	rCent-At (2)	relDist (1)	relDist (2)	relDist (1)[a]	relDist (2)[a]
W<br>C	1	2.449	2.078	-0.466	-0.387	-0.450	-0.371
W<br>C	2	2.302	1.997	-0.421	-0.355	-0.400	-0.334
W<br>C	3	2.633	2.044	-0.471	-0.360	-0.448	-0.337
W<br>C	4	2.035	1.673	-0.353	-0.260	-0.317	-0.225
W<br>C	5	1.426	1.307	-0.163	-0.123	-0.085	-0.045
W<br>C	6	1.349	1.260	-0.142	-0.110	-0.065	-0.033
W<br>C	7	1.547	1.260	-0.197	-0.105	-0.111	-0.018
W<br>C	8	1.332	1.196	-0.135	-0.085	-0.049	0.001
W<br>C	9	1.388	1.235	-0.152	-0.097	-0.067	-0.013
Os<br>C	10	1.095	0.992	-0.041	0.005	0.064	0.110
Os<br>C	11	1.083	1.004	-0.036	-0.001	0.069	0.104
Ru<br>C	12	1.068	0.930	-0.029	0.034	0.081	0.144
Ru<br>C	13	1.048	0.969	-0.019	0.016	0.090	0.126
Ru<br>C	14	1.062	0.885	-0.028	0.056	0.078	0.162
Ru<br>C	15	1.145	1.018	-0.061	-0.007	0.033	0.087
Ru<br>C	16	1.127	1.022	-0.054	-0.008	0.040	0.086

Table S10. Centroid distance ratio and relative to the total (computed using the PM (1) and NLMO (2) π localized orbitals). Relative distance to the *bcp* (a).

At.	Mol.	rCent-At (1)	rCent-At (2)	relDist (1)	relDist (2)	relDist (1)[a]	relDist (2)[a]
W<br>C	2	0.126	0.123	0.831	0.835	0.853	0.856
W<br>C	3	0.186	0.210	0.720	0.685	0.743	0.708
W<br>C	4	0.376	0.404	0.469	0.439	0.504	0.474
W<br>C	5	1.623	1.516	-0.227	-0.196	-0.149	-0.118
W<br>C	6	1.683	1.640	-0.245	-0.233	-0.168	-0.156
W<br>C	7	1.385	1.321	-0.153	-0.131	-0.067	-0.044
W<br>C	8	1.390	1.360	-0.155	-0.145	-0.070	-0.059
W<br>C	9	1.804	1.655	-0.274	-0.236	-0.190	-0.152
Os<br>C	10	1.014	0.934	-0.006	0.032	0.099	0.136
Os<br>C	11	0.934	0.914	0.032	0.042	0.136	0.146
Ru<br>C	12	0.881	0.814	0.058	0.093	0.168	0.203
Ru<br>C	13	0.809	0.797	0.096	0.102	0.206	0.212
Ru<br>C	14	0.844	0.822	0.077	0.089	0.183	0.195
Ru<br>C	15	0.650	0.732	0.198	0.143	0.292	0.238
Ru<br>C	16	0.656	0.656	0.194	0.194	0.289	0.288

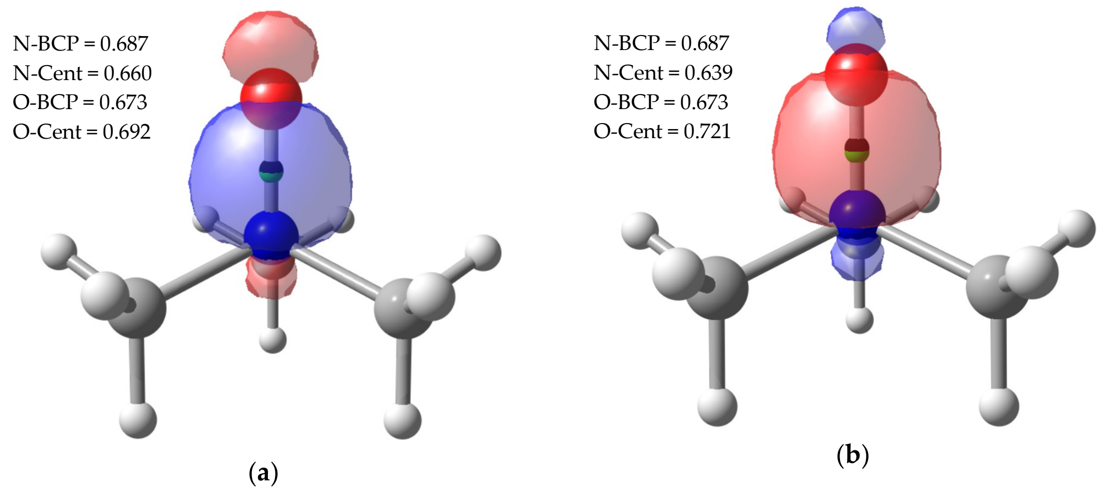

Figure S1. N-O localized σ-type orbitals (PM (a), NLMO (b)) isocontorn plot (0.1) for $(CH_3)_3NO$. *bcp* and centroid represented as black and green dots, respectively. Distances in Å.

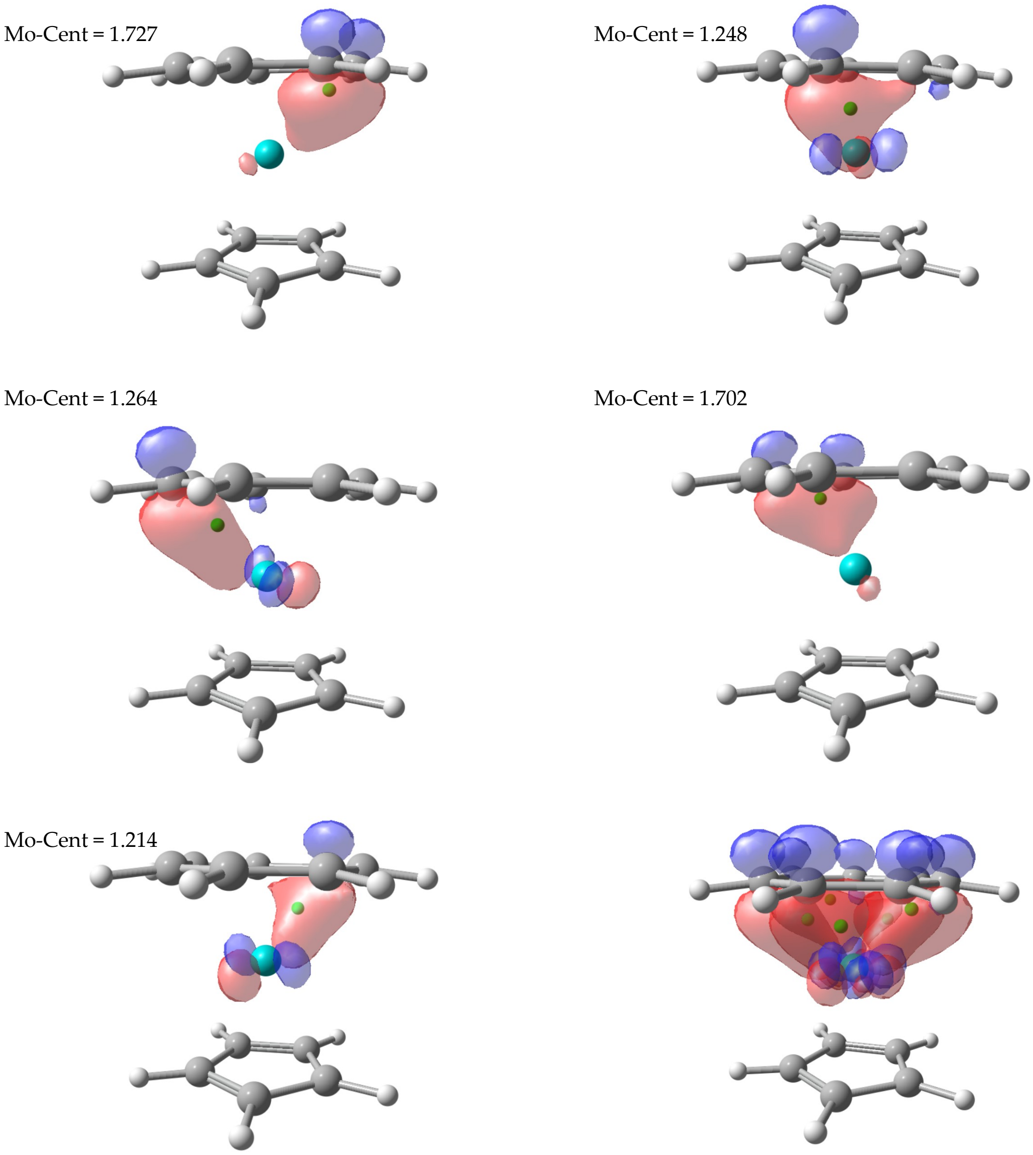

Figure S2. π-type NLMO orbitals isocontorn plot (0.1) for $Mo(C_7H_7)(C_5H_5)$ (a) all together). Centroids represented by green dots. Mo-Ring bonds omitted for clarity. Distances in Å.

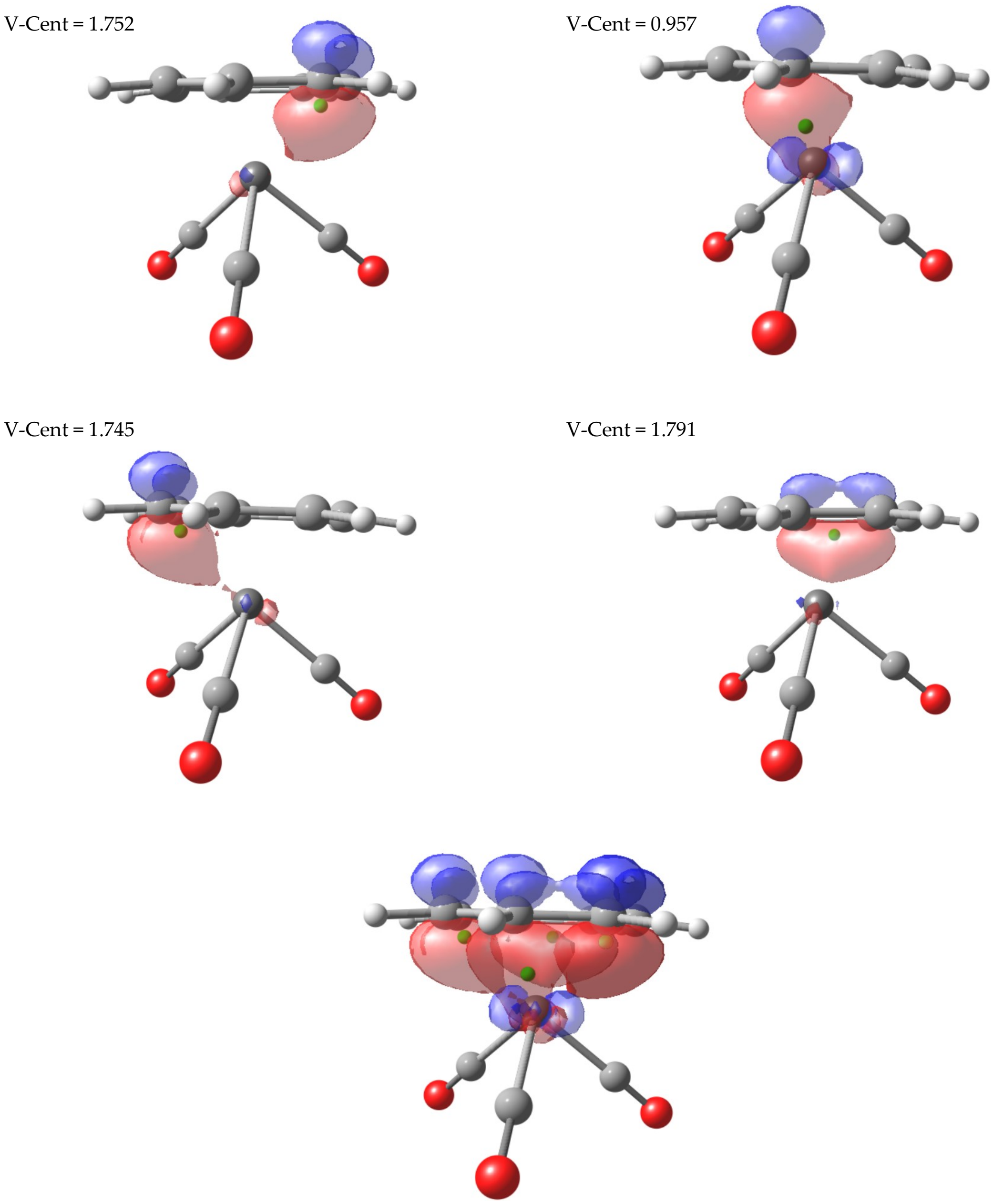

(**a**)

Figure S3. π-type NLMO orbitals isocontorn plot (0.1) for $V(CO)_3(C_7H_7)$ (a) all together). Centroids represented by green dots. V-Ring bonds omitted for clarity. Distances in Å.

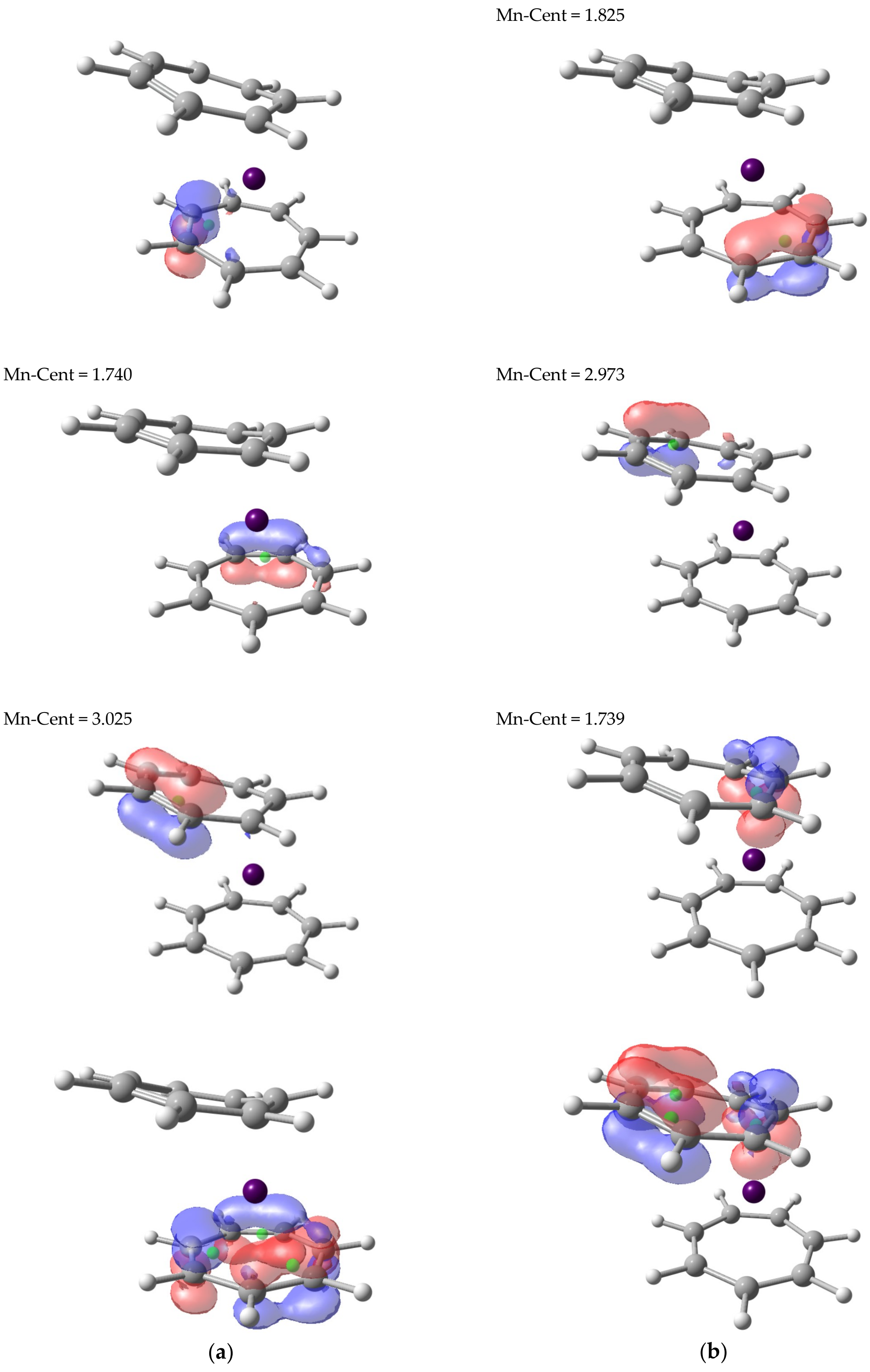

Figure S4. π-type alpha spin NLMO orbitals isocontorn plot (0.1) for Mn(C_7H_7)$_2$ (a) all together η7--type ring and b) all together η3-type ring). Centroids represented by green dots. Mn-Ring bonds omitted for clarity. Distances in Å.

Mn-Cent = 1.553

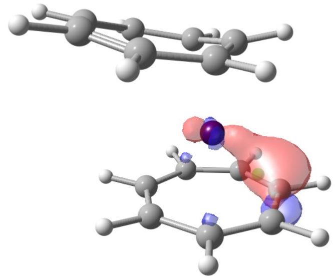

Mn-Cent = 1.819

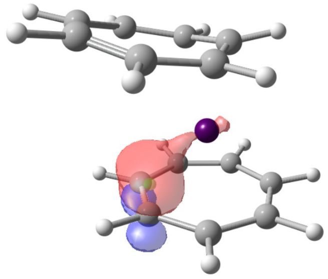

Mn-Cent = 1.821

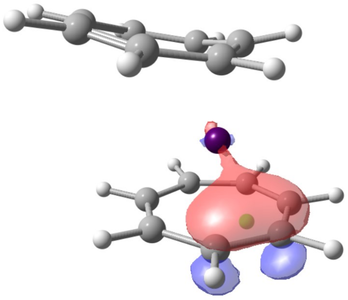

Mn-Cent = 1.821

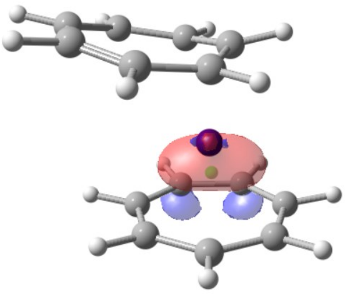

Mn-Cent = 1.165

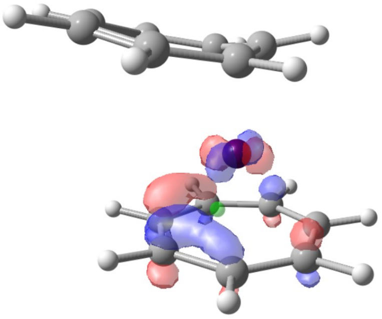

Mn-Cent = 1.290

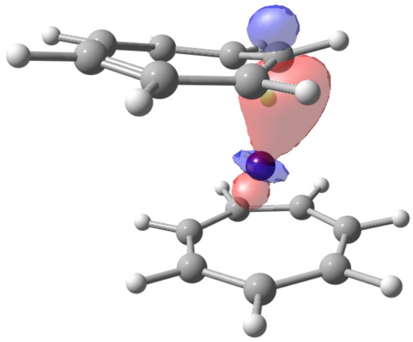

Mn-Cent = 3.277

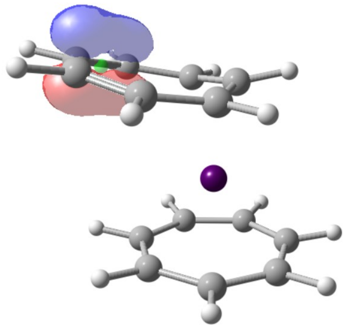

Mn-Cent = 3.277

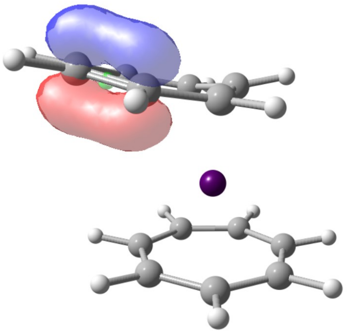

Mn-Cent = 1.698

Mn-Cent = 1.331

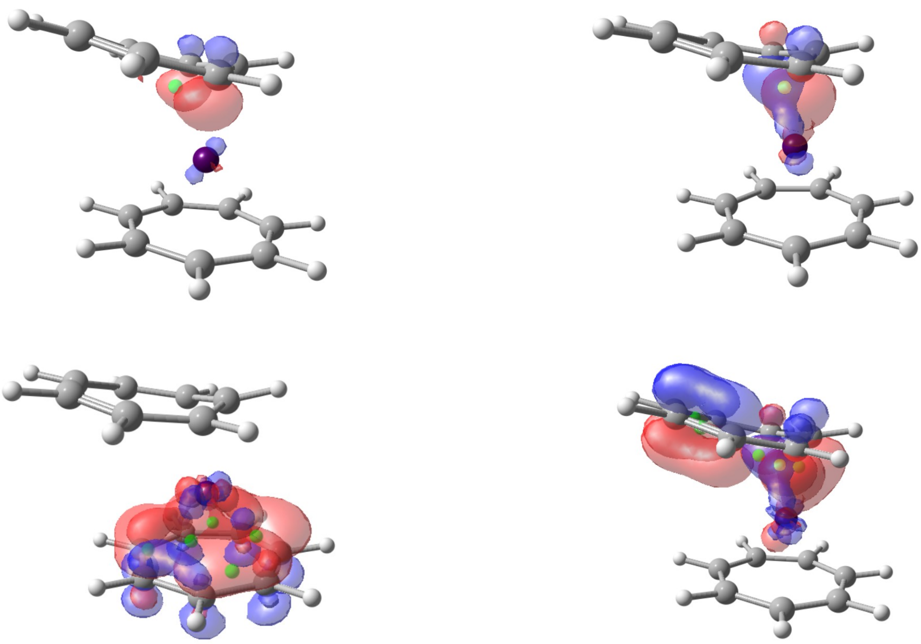

(**a**) (**b**)

Figure S5. π-type beta spin NLMO orbitals isocontorn plot (0.1) for $Mn(C_7H_7)_2$ (a) all together η_7-type ring and b) all together η_3-type ring). Centroids represented green dots. Mn-Ring bonds omitted for clarity. Distances in Å.
